# Citizen Science in Deliberative Systems: Participation, Epistemic Injustice, and Civic Empowerment

**DOI:** 10.1007/s11024-022-09467-8

**Published:** 2022-05-09

**Authors:** Lisa Herzog, Robert Lepenies

**Affiliations:** 1 Faculty of Philosophy, University of Groningen, Oude Boteringestraat 52, 9712 GL Groningen, Netherlands; 2 Karlshochschule International University, Karlstraße 36–38, 76133 Karlsruhe, Germany

**Keywords:** Citizen science, Epistemic injustice, Deliberative systems, Citizen empowerment, Environmental justice

## Abstract

In this paper, we bring together the literature on citizen science and on deliberative democracy and epistemic injustice. We argue that citizen science can be seen as one element of “deliberative systems,” as described by Mansbridge et al. But in order to fulfil its democratic potential, citizen science needs to be attentive to various forms of exclusion and epistemic injustice, as analyzed by Fricker, Medina and others. Moreover, to tap the potentials of citizen science from the perspective of deliberative democracy, it needs to move towards a more empowered approach, in which citizens do not only deliver data points, but also, in invited or uninvited settings, participate in discussions about the goals and implications of research. Integrating citizen science into the deliberative systems approach embeds it in a broader framework of democratic theory and suggests the transmission of certain practical strategies (e.g., randomized sampling). It can also contribute to realism about both the potentials and the limits of citizen science. As part of a deliberative system, citizen science cannot, and need not, be the only place in which reforms are necessary for creating stronger ties between science and society and for aligning science with democratic values.

## Introduction

Citizen science is a movement that aims at involving citizens in research projects. Originally coined by Irwin (1995) and Bonney (1996), there are now various official definitions of the term, which all focus on the collaboration between lay people and scientists, often with the aim of addressing real-life problems (see Hecker et al. 2018a; Strasser et al. 2019; Schrögel and Kolleck 2019 for overviews and categorizations). While it has a long history, especially in wildlife observation, citizen science has seen an upswing in recent years, with projects being undertaken in numerous countries.^1^ Projects take on a wide variety of forms: Citizens not only report about environmental changes (e.g., counting insects), they also measure air quality, or contribute astronomical observations. Some researchers have high hopes for the potential of citizen science, not only with regard to scientific insights, but also with regard to increases in scientific literacy and civic engagement, and the building of trustful relationships between science and society (e.g., Bonn 2020: 14). In 2018, researchers from the European Citizen Science Association developed *Ten Principles of Citizen Science* (Robinson et al. 2018), which emphasize, among other things, that citizens should have a “meaningful role” in projects, that the aim is to generate “genuine science outcome[s],” that there is supposed to be mutual feedback and acknowledgment of all contributions, and that the results should be made publicly available. Indeed, citizen science is invoked to potentially tackle the grand current and future challenges, from Covid^2^ to global sustainability.^3^

But are these expectations justified—or is citizen science just a pleasant hobby for highly educated people, or a handy way for scientists to gather unwieldy data? To answer this question, and to see how the potentials of citizen science could best be unlocked, we suggest situating citizen science within the framework of deliberative systems (Mansbridge et al. 2012; see also Bächtiger et al. 2018). The vision of deliberative democracy emphasizes the role of argument-based discussions between citizens, in which political issues are articulated and opinions and preferences are formed. Within this line of theorizing, the deliberative systems approach understands deliberation as distributed over the different elements of a “deliberative system.” It includes forums of deliberation that range from everyday conversations between citizens, to conversations in social movements or associations, media discussions, exchanges in newspapers, and parliamentary debates (Mansbridge et al. 2012; Owen and Smith 2015). By drawing on this theoretical framework, we can put citizen science into a broader perspective and connect it to current debates in democratic theory.^4^ Citizen science has much democratic potential—a deliberative systems approach shows how the democratic potential of citizen science can be leveraged.

Understanding citizen science within the broader conceptual framework of deliberative systems helps to articulate some of the hopes—for enlightenment, for greater trust in science, for more rational discourses—that have been connected to citizen science, but also to see some of the limitations of citizen science, especially of “invited” or “scientific-authority driven” formats (see Wynne 2007; Wehling 2012 on the former and Ottinger et al. 2017 on the latter distinction). From the perspective of deliberative systems, it is quite interesting to note what the *Ten Principles of Citizen Science* (Robinson et al. 2018) do *not* discuss: for example, which kinds of topics should be prioritized (and whether citizens should have a say in this), the connection to democratic practices more broadly speaking, the question of *which* citizens to involve, the question of how citizen science projects relate to political actors and/or social movements, the extent to which projects should be open to discuss normative and political questions at the intersection of science and society, or the question of which bigger vision for the relation between science and society underpins citizen science.

Many practitioners and theoreticians of citizen science certainly do have answers to these questions. But we take it that by bringing the reflections on citizen science into dialogue with other strands of literature can help better articulate these issues, and thereby move the debate forward. Therefore, our aim in this paper is to draw on the tools of the deliberative systems literature, together with the literature on epistemic injustice (e.g. Fricker 2007; Medina 2013) and various literatures on participation, empowerment and co-creation (Lorenz 2020; Pandya 2012; Chevalier and Buckles 2019; Chilvers and Kearnes 2019), in order to make them explicit. We thereby also respond to the call to introduce more reflexivity in citizen science and to engage in a dialogue with the social sciences and humanities (Mahr et al. 2018; see also Strasser et al. 2019).

Our approach thereby aims at discussing the *democratic* potential of citizen science. But from that perspective, we also need to ask critical questions about the ways in which citizen science projects are run: Which subgroups of citizens are involved? Are the projects structured such that democratic values, in particular inclusivity and epistemic justice, are taken into account? Are citizens taken seriously as equal partners, who might also contribute to decisions about the goals and strategies of research? Are societal questions (e.g., of social justice), which go beyond the scope of data gathering, addressed? The sensitivity to such questions can help practitioners of citizen science who have wider democratic aims to design their projects accordingly. However, the deliberative systems perspective also makes clear that one should not expect citizen science to be a magic wand that could, on its own, bring about transformative change. Rather, it should be seen as going hand in hand with other reforms, within the deliberative system as a whole, which would improve the relation between science and society and bring science in line with democratic values. In times in which climate skepticism, anti-vaccine movements, and conspiracy theories boom, citizen science, if done well, can potentially be an important element of strategies that aim at strengthening a positive relation between science and society. But it cannot, and need not, replace reform in other parts of the deliberative system.

In the next section (II), we elaborate on our proposal to see citizen science as one element within the context of deliberative systems, emphasizing the role of transmission mechanisms between different elements of such systems and the need for context-sensitivity. We then argue (section III) that in order to live up to its democratic potential, citizen science needs to be attentive to general questions about inclusivity, but also specific questions about epistemic injustice or epistemic oppression. Our third claim (section IV) is that from the perspective of deliberative democracy, citizens need to be treated as equal partners in deliberation, which implies that their role cannot be only to deliver data points, but must also include the possibility of asking questions and putting forward proposals for the goals and strategies of research, and of reflecting and arguing about the underlying societal issues and policy questions. This often happens automatically in “uninvited” or “social movement-based” (Wynne 2007; Wehlinger 2012; Ottinger et al. 2017) forms of citizen science—which thus deserve a certain priority from the perspective of deliberative systems—, but should also be systematically integrated in “invited” or other forms.

These three arguments, taken together, provide an account of the role that citizen science could, potentially, play in the deliberative system of a democratic society. However, we also caution that these expectations should not be understood as realizable in each and every citizen science project. We argue that citizen science should, instead, be understood as one element among many within a deliberative system, and that it should not be overloaded by expectations that could only be fulfilled by broader reforms (section V).

## Citizen Science Within Deliberative Systems

Deliberative democracy is a branch of democratic theory^5^ that emphasizes the exchange of arguments and ideas between citizens. Building on earlier work by Jürgen Habermas, John Rawls and others, it is now a flourishing branch of political theory (for overviews and important contributions, see e.g., Bohman 1998; Dryzek 2000; Chambers 2003; Gutman and Thompson 2004; Parkinson 2006; Goodin 2008; Bächtiger et al. 2018). Instead of focusing exclusively on voting, which aggregates the preferences of citizens, deliberative theory takes into account the *processes* in which political preferences are formed, emphasizing the role of discourse. In private and public discourse, citizens come together and exchange their different perspectives, articulating problems that need to be addressed and forming their opinions and preferences. When these conversations fulfil certain criteria, such as mutual respect, equal standing, the non-strategic use of arguments, honesty, and accountability, they can be described as “deliberation” (Bächtiger et al. 2018). As such, deliberative theory has certain commonalities with “epistemic” theories of democracy, which emphasizes the ability of democratic processes to come to good solutions, because multi-perspectival and participatory decision-making is more likely to discover all relevant aspects, respond to all relevant objections, and explore all options, than decision-making by single individuals or small groups (see e.g., Landemore 2012).

Deliberative theory has been a source of inspiration for various social experiments. For example, researchers use “deliberative polls” for bringing together randomly selected citizens to “deliberate,” by help of facilitators and with the possibility of asking technical experts for advice on specific topics (see e.g., Fishkin and Luskin 2005; Fishkin 2018). But such experiments can obviously only involve a small fraction of the citizens of a society. In fact, an open question for the earlier, more theoretically-focused contributions to democratic theory was where deliberation actually took place in practice. Moreover, critics (e.g., Fraser 1990) had questioned the focus on rational arguments, at the exclusion of emotions, narratives, and utterances of legitimate interests. They also raised the point that “the democratic public” can and should be understood as a multiplicity of publics. This made it all the more pressing to ask about the space—or rather: spaces—in which deliberation actually takes place in democratic societies.

The “deliberative systems” approach attempts to provide answers to these questions (Mansbridge et al. 2012; see also Owen and Smith 2015; the first use of the term was in Mansbridge 1999). According to this approach, the places of deliberation are many: they include public discourse in the sense of media and social media, discourse in parliaments and other public institutions, discussions in NGOs and social movements, but also citizens’ everyday talk among themselves.^6^ All these elements take place in material, social and cultural settings that have an impact on how well deliberation can (or cannot) function. The different elements are connected, and each can, potentially, contribute to the deliberative quality of the system. But not *all* parts of the system need to be deliberative (Mansbridge 1999: 224). Sometimes, institutions might have constraints stemming from their functions that do not allow for deliberation (Mansbridge et al. 2012: 12–13); sometimes non-deliberative forms of communication (e.g., street protests) can also contribute to the overall quality of the deliberative system, for example by drawing attention to neglected issues (Mansbridge et al. 2012: 6, 17–19). What matters, overall, is that three basic functions of the deliberative system are fulfilled: an *epistemic* function of processing information and exchanging arguments; an *ethical* function that promotes relationships of respect between citizens, and a *democratic* function that ensures the inclusion of various interests and concerns (Mansbridge et al. 2012: 10–13).

We suggest embedding citizen science into the perspective of deliberative systems. Citizen science combines scientific aims (the generation of scientific knowledge) and the broader public discourse (by involving members of the general public). It can take the form of “invited” citizen science, initiatives by universities or public institutions, or of “uninvited” initiatives, often driven by social movements or groups of activists (Wynne 2007; Wehling 2012; for discussions of the latter, see e.g., Ottinger et al. 2017; Kimura and Kinchy 2019).^7^ As such, it can be a strategy for addressing the risk of a “decoupling” of different parts of the system, namely, between scientific and public discourse. Mansbridge et al. (2012: 23) describe such “decoupling” as one possible defect of deliberative systems (others are “too tight coupling,” which makes critical feedback impossible, institutional, or social domination, and extreme partisanship, ibid.,: 22–24).

Adopting the perspective of deliberative systems means emphasizing that through citizen science, the responsibility of science to society, which was one of the original impulses of citizen science (Irwin 1995), could be strengthened, while citizens could benefit along epistemic, ethical, and political lines. This is how citizen science is in fact often understood. As one group of authors puts it (Bonn 2020: 10): “Citizen Science provides the possibility to move towards educational concepts for society as a whole, that aim at strengthening key competences for a successful life and a functioning society.”

Citizen science can contribute to the three functions Mansbridge et al. describe. On the *epistemic* side it not only generates knowledge and points to neglected areas of research or “undone” science (Frickel et al. 2010), but also helps citizens to understand how scientific results are generated, making them more competent to evaluate evidence and to form their opinions. It might also contribute to empowering *groups* to play a more active epistemic role within a deliberative system, for example, by supporting grassroots movements and social movements to produce scientific results (Mahr et al. 2018: 99; for examples, see Brown 1992 on “popular epidemiology,” Wehlinger 2012: 49–50 on patient-driven medical research; Ottinger 2010 on the “Louisiana bucket brigade”; see also Landström 2020 and Kimura and Kinchy 2019 for various examples in environmental governance).

With regard to the *ethical* function, it can help overcome a picture in which scientists are authorities to which lay people must bow, and instead foster relations of “mutual respect among citizens” (Mansbridge et al. 2012: 11). As Hecker et al. (2018a: 7) put it, “Research is literally ‘opened’ up to members of society and they often become part of the whole process, thus making science more inclusive.”^8^ This can help overcome a hierarchical, or antagonized, relation of “us” versus “them” and create a “we” between researchers and citizens.^9^ As such, it can be one of the “technologies of humility” for scientists that Jasanoff (2003a) has called for.

This ethical outcome might, in turn, have beneficial epistemic effects for the deliberative system as a whole (see similarly Bonn 2020: 16–17). This is the case because it can be a mechanism for establishing a trusting and trustworthy relationship between citizens and (credentialed) experts: it can not only create bonds of solidarity, e.g. when researchers and citizens join forces to address an environmental challenge, but also helps to create “overlapping understanding,” when citizens become (partial) experts in some area and can communicate their knowledge to others (on these mechanisms, see Christiano 2012: 37–38).

Lastly, with regard to the *democratic* dimension, at least some citizen science projects can also make a contribution in this respect (while others may not have an immediate relation to any political concerns). As Mansbridge et al. write (2012: 12), “The inclusion of multiple and plural voices, interests, concerns, and claims on the basis of feasible equality is [...] the central element of what makes deliberative democratic processes democratic.” Such concerns can, for example, come from policy-relevant areas such as public health or environmental protection, where “uninvited” or “bottom-up” initiatives can help close knowledge gaps. Many citizen science projects do indeed take place in such areas, e.g. when it comes to biodiversity questions; below we will emphasize the benefits of collaborations with community groups on such issues.

Looking at citizen science through the lens of the deliberative systems approach also helps to see important conditions for it to be successful. An important question is how citizen science projects are connected to other deliberative forums, such that relevant information can be transmitted. Without such transmission mechanisms, the results of projects cannot lead to societal or political change. This, in turn, is likely to lead to frustration and resignation among the participating volunteers in the long run.^10^ Important mechanisms of transmission take place in the media, whether local or otherwise. This means that it often makes sense to combine citizen science projects with strategies for science communication (see e.g., Hecker et al. 2018b). Another possibility is the direct connection to policymakers, again both on the local and on other levels. These transmission mechanisms vary, of course, from country to country.

This is a point that many writers emphasize: citizen science projects can help address local problems, involving all stakeholders in the process of finding solutions (e.g., Bonn et al. 2020: 10; Novak et al. 2018: 126; Nascimento et al. 2018).^11^ In this way, citizen science can be seen as contributing to both the co-production of public services and the co-production of knowledge for improved decision-making (Miller and Wyborn 2018; Lepenies et al. 2018). Some authors—particularly in the sustainability sciences—have praised it as a strategy for the ecological transformation, which cannot succeed when implemented in a top-down, technocratic fashion, without drawing on the local knowledge and expertise of citizens (e.g., Fischer 2000; Volten et al. 2018; Owen and Parker 2018; Dosemagen and Parker 2019).

For this strategy to be successful, and for unlocking the potential of citizen science within a deliberative system, however, two conditions need to be fulfilled: (1) citizen science projects must be *conducted* in a way that can serve these aims while also upholding the ethical commitment to democratic equality; (2) other elements of the deliberative system—and indeed of the political system in a more general sense—need to be able to receive the knowledge, impulses, and proposals that come from citizen science initiatives, i.e., the “coupling” must function well and there must be no institutional or social dominance. While fulfilling the first condition is, to some extent at least, in the hands of the organizers of citizen science projects, they usually have limited influence on the second. In the next two sections, we discuss two requirements for the first condition; in the last, we conclude by discussing why a realistic take on the potentials of citizen science within deliberative systems is needed.

## Citizen Science, Inclusivity, and Epistemic Injustice

In order to unlock the potential of citizen science as one element of deliberative systems, the three functions that Mansbridge et al. (2012) describe—epistemic, ethical, and democratic—need to be kept in mind. To realize these functions, two normative principles can be distinguished: a general notion of inclusivity of citizen science towards *all* members of society, and a more specific notion of “epistemic justice” that aims at avoiding violating individuals’ standing *as knowers* (Frickel et al. 2010; Medina 2013).

When new forums for deliberation are opened up, a key question is whom they include and thereby enable to speak, and how reinforcements of existing biases can be prevented. But at least in certain citizen science projects in invited formats, there seems to be a lack of inclusivity along demographic lines. Take, for illustration, two ornithology citizen science projects that Edwards et al. (2018: 385) report on, in which “83 per cent of respondents were male, 98 per cent were white and the largest proportion was in the 61–70 age range” with 67 percent of respondents having university-level qualifications. Citizen science projects can run the risk of comprising precisely “those citizens who probably already have the most resources to engage in policy in the first place (e.g., time, capital)” (Nascimento et al. 2018: 233). The participation of predominantly higher socioeconomic strata in invited citizen science projects is a well-established finding (Haklay 2018, citing Curtis 2015; Raddick et al. 2013; Budhathoki and Haythornthwaite 2013; Causer and Wallace 2012). Haklay notes: “Across projects, the participation of people with tertiary education is at least twice the level in the general population, and the participation of people with doctoral-level education is at least three times higher” (Hakley 2018: 56).

One might ask how much of a problem is, if, for example, projects in astronomy are done mostly by white men. But from the perspective of deliberative systems, there is indeed a loss, in the sense that it is a missed opportunity for democratic integration and for individuals from different backgrounds to meet each other as equals. Moreover, citizen science projects that mainly attract highly educated white male individuals reproduce stereotypes about who does and does not belong in the social space of science, thereby contributing to the ongoing mechanisms of exclusion in these fields (on stereotype threat, see e.g., Goguen 2016, on exclusion from academia as a form of epistemic justice, see Hänel 2020).

The need for greater inclusivity in citizen science activities has been noted in the literature (Richter et al. 2018: 282). Many citizen science organizers are expressly attempting to make science more “inclusive across gender, ethnicity, class, disability, level of education” and age (Makuch and Aczel 2018: 395) and argue that “underserved communities and unheard voices need to be included in a people-powered science” (Nascimento et al. 2018: 233; see also Leach et al. 2005). Seeing citizen science as an element of deliberative systems can help to think about possible strategies.

A first, obvious step is to monitor the composition of citizen science projects in terms of gender, ethnic, socioeconomic, and educational attainment, which so far often does not mirror general population characteristics (and it might also be helpful to collect date about the characteristics of those *running* citizen science projects, as a general lack of diversity in science may be transposed into a lack of diversity in citizen science^12^).

Secondly, taking the systemic character of deliberative democracy into account, a useful strategy could be to tie citizen science projects to institutions or forums that are more inclusive. For example, if there are inclusive public schools in a community, these can serve as anchor point for reaching not only children from different backgrounds, but also their parents. Another strategy might be to organize citizen projects at workplaces, because these are often places in which individuals from different ethnic and ideological backgrounds (though not always from different socio-economic backgrounds) meet (Estlund 2003).

Lastly, a direct inspiration from projects in deliberative democracy and democratic experimentalism could be to introduce lottocratic elements, i.e., to draw potential participants by lot from all strata of society (on the benefits of lottocracy, see e.g., Guerrero 2014). As the experiences of setting up “citizen assemblies” or “deliberative minipublics” show, recruiting democratically diverse samples can take some efforts. But once a broad range of citizens participates in discussions, existing evidence shows that the opinions of male, privileged participants do not disproportionately influence the outcomes of debates (Fishkin 2018: 76–77), probably as a result of the careful facilitation and moderation of these processes. This is a practice that citizen science projects could also adopt; ideally, practices that have been proven successful (e.g., certain strategies in recruiting participants) should be made publicly available, so that other organizers can learn from them and adapt them to their own context.

In addition to the general principle of inclusivity, citizen science projects must also avoid more specific epistemic injustices. Fricker (2007) has introduced the term “epistemic injustice” for describing forms of injustice that concern individuals in their role *as bearers of knowledge* (see also Egert and Allen (2019) and William and Moore (2019) on “knowledge justice”). One category of epistemic justice that is relevant in for citizen science is “testimonial injustice,” which occurs when individuals who are categorized as female or non-white are taken less seriously and their testimony is discounted compared to that of individuals from more privileged groups (Fricker 2007, chap. 2).^13^ Such injustices can be observed in personal interactions or institutional contexts (e.g., in court proceedings), but they need to be understood against the background of broader societal injustices along lines of gender, race, and socio-economic status. In such structures, privileged individuals can often afford to remain ignorant of problems that concern mostly disadvantaged individuals, and they can afford to keep blind spots they are not even aware of (what Medina (2013, chap. 2.3) calls “meta-blindness”). Moreover, the access to epistemic resources is distributed in unequal ways, again along similar demographic lines, which can lead to “epistemic oppression,” the exclusion of certain groups from the processes in which knowledge is generated (Dotson 2014; Polhaus 2020).

What is particularly relevant in the context of citizen science is the risk of violations of epistemic justice towards individuals who have something specific to contribute—members of what Michaels (2009: 623) calls “publics in particular” who have a stake in certain issues. Fricker explains “testimonial injustice” as based on “identity prejudices,” i.e. stereotypes about which groups do or do not deserve to be heard (Fricker 2007, chap. 2). As Fricker emphasizes throughout her work, epistemic injustices are moral *and epistemic* failures, because they leave out the voices of certain individuals or groups. As authors in the literature on the epistemic benefits of democracy (e.g., Landemore 2012) emphasize, one of its greatest potentials is to provide multi-perspectival discussions and to uncover blind spots that people (and in particular, non-democratic ruling elites) might have. But epistemic injustices block this potential, hence there are also instrumental reasons to try to avoid it.

This is of particular importance in cases in which the nature of the policy problem (e.g., an unstructured problem that requires learning processes with multiple feedback loops), suggests that participation is needed (cf. e.g. Hurlbert and Gupta 2015 on the “split ladder of participation”). Many policy problems require building trust between different groups (Hurlbert and Gupta 2015: 103), including scientists, but also including experts with “local knowledge,” as famously illustrated in Wynne’s study of “sheepfarming after Chernobyl” (Wynne 1989). But epistemic injustice undermines the possibility of building trust, because, as Fricker explains, it undermines individuals in their “capacity to give knowledge to others,” which “is something that can cut deep” (2007: 44).

While such stereotypes and the ensuing epistemic injustices can refer to individuals *qua* women, non-whites, etc., they can also concern their very status *as lay people.* By including them in citizen science projects, scientists of course want to give them a voice (overcoming yet more unjust, older structures in which lay people were not seen as worthy of any form of inclusion). Nonetheless, there are remaining questions about the *role* that “citizens” play in “citizen science,” and how to evaluate them from a deliberative systems perspective. We turn to these in the next section.

## Empowering Citizens in Citizen Science

To tap the potentials of citizen science from the perspective of deliberative democracy, citizen science needs to move towards a more empowered approach for citizens, in which citizens do not only deliver data points, but also participate in discussions about the goals and implications of research. In the literature on citizen science this has been discussed as the move from treating citizens as “data drones” towards fuller forms of participation, and even co-productionist engagements.

Many commentators call for strengthening citizens’ voice in citizen science projects (see English 2018 on “extreme citizen science” or Haklay (2018) on “collaborative science”). Bedessem and Ruphy (2020) helpfully distinguish between contributory citizen science, collaborative citizen science and co-created citizen science as three main ways to engage citizens with increasing levels of co-determination, whereas Nascimento et al. (2018) documents different participatory models in CS projects (bottom-up vs. top-down, with the latter far more prevalent; see also Leach et al. 2005). Citizen science projects can, potentially at least, also lead to a broader dialogue about the way science functions and the role it places in society: “citizen science can challenge the ways scientists produce knowledge, including their assumptions and standards about what is valid as scientific knowledge” (Nascimento et al. 2018: 235).

From this perspective, one can even argue that priority should be given to “uninvited” forms of citizen science (e.g., Brown 1992; Wehlinger 2012; Wynne 2007; Ottinger 2010; 2017; Kimura and Kinchy 2019; Landström 2020). In them, citizens are in the “driver’s seat” (Christiano 2012) from the start, often in the form of social movements or associations that form around a certain issue. As Ottinger notes (2017: 352), they start from a problem, and “in order to compel authorities to recognize and address the issues, they marshal information—monitoring data, health data, geographic information—that can serve as evidence of the problem and point to its sources.” In this way, blind spots and “undone science” (Frickel et al. 2010) can be discovered and the implicit assumptions on which existing research programs are built can be challenged. As Wynne (2007: 107) puts this point:

[D]eliberately or not, *invited* public involvement nearly always imposes a frame which already implicitly imposes normative commitments—an implicit politics—as to what is salient and what is not salient, and thus what kinds of knowledge are salient and not salient’ … ‘[U]ninvited forms of public engagement are usually about challenging just these unacknowledged normativities’

In a similar vein, Kimura (2021) argues that the relation of citizen science to social movements should be understood as “multi-actor,” “process-oriented” and “long-term,” and that it can unleash new potentials for questioning existing value structures and patterns of thought.

It is interesting to see, in this context, what strategies are used in environmental justice movements (Blake et al. 2020; Bullard et al. 2008) and by other activist groups that utilize community science methods, putting knowledge into action in alignment with the interest and goals of underserved communities (as detailed by Lorenz 2020). Environmental justice movements and similar communities understand themselves as engaging in particularly problem-driven activities. They frequently highlight the unequal burdens from pollution (e.g. in terms of health) that marginalized communities experience—yet often utilize scientific methods and evidence themselves (e.g., when monitoring pollution; when mapping impacts of environmental hazards on socioeconomic group).Whether as participant action research, engaged scholarship, or community science or environmental justice groups, various groups have long undertaken activities that could be classified as citizen science (though without calling it such).

*Invited* forms of citizen science can learn from these experiences and try to integrate strategies for empowering citizens, in combination with efforts to fight against epistemic injustice. One strategy is to introduce antagonistic elements into research projects, in order for meaningful debate to occur where participants can balance their roles as scientific investigators and citizens. Here, the literature on deliberative democracy can help by suggesting tools and frameworks for how this can be discussed (Bächtinger et al. 2018). Such antagonistic elements can be found in fields of scholarly engagement that are very close to citizen science, but have been remarkably rarely absorbed, such as the engaged scholarship (Raphael 2019) or the environmental justice literature (Holifield et al. 2017) as well as participant action research (Chevalier and Buckles 2019). An exception to this are select projects that have explicitly attempted to decolonize citizen science, for example, through community-based monitoring projects (Cohen et al. 2021; Bhawra 2022).

Another strategy for “invited” forms of citizen science is to aim at engagement with civil society actors or social movements; funders should acknowledge and support such alliances (as some already do). This could bring improvements with regard to inclusion and epistemic justice, as discussed in the previous section, and avoid turning such projects into “experiments with citizens” that are cut off from real-life controversies and driven mostly by expert agenda setting (Bogner 2012). Indeed, a main reason for lack of diversity and engagement is seen by Pandya (2012: 314) in the fact that there “may be an absence of alignment between community priorities and research objectives.” We concur with Pandya that many citizen science projects could benefit by actively building on community priorities. Similar calls to “social” citizen science have been brought forward, which acknowledge the close relation between scholarship that fails to address social justice issues as well as lacks diversity of participants (Lorenz (2020: 2) proposes a model of social citizen science that “utilizes a community-based research framework to bring together *social scientists* and publics to solve” real world problems).

From the perspective of deliberative systems, which emphasizes the need for “coupling” between different discursive spheres, it is also desirable that citizen science projects go beyond immediate concerns that require policy interventions or regulation (e.g., with regard to the use of nano technologies) and ask more general questions about the relation between science and society, using more concrete questions and examples as starting points. This could be an explicit goal of citizen science projects. In other words, we argue for wider political and discursive goals to be included (and funded) as part of citizen science. Here, citizen science can learn from the reflexive ambitions of science and technology studies and science studies. Chilvers and Kearnes (2019: 360–3), for instance, talk about “responsible democratic innovations” (taking up the principles of responsible research and innovation), which concern, for example, the ethical implications of research or reflection on evaluation criteria, all while acknowledging that these topics are complex and controversial, and may require case-by-case assessments. Such broader reflection upon citizen science projects would bring citizen science projects closer to deliberative ideals.

As Kimura and Kinchy (2019: 141) point out, citizen science can also help to counter the increasing influence of private money that sets the agenda in research. With public research budgets being cut in many countries, universities and other research institutions increasingly rely on money from corporations or philanthropists, which raises serious questions about the biases this can introduce. In contrast, “[*c]itizen*-based science can help diversity the perspectives and experiences that inform research” (Kimura and Kinchy 2019: 141). To bring in various voices, Shapiro et al. (2017: 586) suggest a strategy that they describe as “inviting apprehension,” which tries to include many different perspectives and to articulate “the question before the questions,” (ibid.), e.g., by using artistic forms of expression in order to engage in “mediating, re-situating, or re-imagining engagement and discussion” (ibid.: 596).

Such moves towards a more applied and community-oriented form of citizen science have also received prominent criticism. A recent comment in *Nature* raises objections against a “politicization” of citizen science (Nature 2015). The worry is that participants in citizen science projects might pursue political objectives; for example, “opponents of fracking [...] might help track possible pollution because they want to gather evidence of harmful effects” (Nature 2015). The picture that seems to stand in the background of this commentary is one where the pure pursuit of scientific goals is polluted by the intrusion of political or other values. However, this view has long been refuted in the philosophy of science, where the importance of values in the scientific process but also their careful delineation to specific roles, have long been discussed (Douglas 2009; Kitcher 2011).

We would argue, to the contrary, that it is a *lack* of politicization, and a bypassing of normative (and hence political) questions, that is problematic (see similarly Leach et al. 2005: 7–9; Jasanoff 2003b; cf. also Kimura and Kinchy 2019: 4, 138 on “taking a stand”). The picture suggested by the *Nature* comment might seem justified if one based it on the assumption that scientists had a better normative compass than citizens, and should therefore determine the goals and purposes of the research project on their own. But this is a highly problematic assumption, given that scientists are no better trained in answering normative questions than citizens. One might even argue that the dysfunctional incentives within academia might distort their judgment, e.g., about the relative importance of different research areas (e.g., Sarewitz 2016; Edwards and Roy 2017). As Wehling points out (2012: 45), public participation can be “both more effective and legitimate” if the groups who are involved put their interests and values on the table.

From within the citizen science community, there are increasing calls to “take up questions from society on real-life problems” and “involve citizens in research processes relating to society at an early stage and on an ongoing basis” (Bonn 2020: 13). The unavoidable frictions that come from such activities, including charges of “politicization,” should not be seen as an unwanted byproduct, but rather as a sign of healthy deliberation, aided by scientific tools, about the very question of what constitutes relevant knowledge for society. Practitioners of citizen science in this vein also need to be aware, however, of the various paradoxes and unintended consequences that they can imply. Ethnographic accounts of citizen science projects and their interaction with politics (e.g., Zilliox and Smith 2018) show that there can be unintended consequences and unexpected turns, because the perceptions of all groups involved (both of each other and of the topics) can shift. An example explored by Shapiro et al. (2017: 580) is that the very pursuit of data can lead to a situation in which access to data becomes a precondition for participating in discussions. There is no “one-size-fits-all” solution for such dilemmas and challenges; instead, citizen science projects need to plan for integrating time for reflection, and reflexivity about their own activities, from the start (see also Kimura and Kinchy 2019: 23).

With such an approach, citizen science would treat participants not just as non-credentialed scientists, but as veritable experts—namely, as experts in democracy. Citizen science projects designed along these lines could become flourishing sites of deliberation. To be sure, they cannot, and should not, replace democratic politics. Certain deliberations need to take place in ways that involve *all* citizens, not only those involved in community organizations, environmental justice movements, or the citizen science projects that cooperate with them. But well-designed, participatory citizen science projects nonetheless have the potential to support the overall quality of the deliberative system, especially if they throw light on unjustly neglected problems and bring together various forms of expertise that are all needed to address certain issues, but which all too often fall into the cracks between different social spheres or are neglected for lack of credentials.

## Realistic Expectations for Citizen Science

So far, we have discussed two sets of issues that arise if one thinks of citizen science as one element of deliberative systems: questions about inclusivity and epistemic justice, and questions about how to ensure that citizens are empowered and the practices of citizen science serve an ideal of reflexivity, which includes discussions about the goals and values of research. Table 1 summarizes the concrete proposals that flow from this perspective. The perspective of deliberative systems also helps, however, not to overburden citizen science with unrealistic expectations. Given its potentials, there is a risk of expecting too much from citizen science: more public trust in science, better democratic discourses, more involvement of minority groups, etc. But from the deliberative systems perspective, two important points can be made, which can help to keep the expectations for citizens science realistic: (1) citizen science is one strategy among many to improve deliberation, but it cannot and need to be the only one; (2) there is a “division of labor” between different deliberative sites, and not all projects need to reach all goals.

**Table 1:**
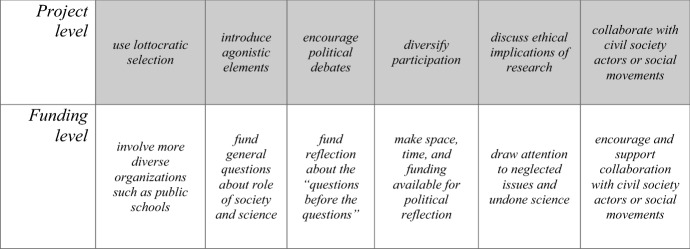
Proposals for strengthening the democratic dimension of citizen science at the levels of projects and funding

The first point easily comes into view when one takes into account the way in which deliberation is *distributed* over many social spaces in a deliberative system. Not all of these spaces need to be (fully) deliberative; it is the overall quality that matters. However, one important question concerns the risk of a “decoupling” of different parts of the system (Mansbridge et al. 2012: 23). Earlier, we have emphasized the need for transmission mechanisms that make sure that the results of citizen science projects are made more widely known. For example, are there associations or social movements with whom scientists can collaborate, but who can then also carry the results of study further and push for change? Or relatedly: can the engagement of volunteers be maintained in constellations in which data collection is anticipatory (i.e., *not yet* transmitted to anyone) and might in fact never be needed?^14^

Another example is media uptake: are there media that can report about the results of citizen science projects and bring the underlying issues to the attention of a broader public and politicians? While some citizen science projects have included a link to the media from the start (see Hecker et al. 2018b for examples), this may not be easily possible if there are no local newspapers (on the dearth of local newspapers, in the US, see e.g., Teachout 2020, chap. 3), and many existing media channels are dominated by commercial interests.^15^ There have been various calls for reform in the media system, to align it better with democratic needs (Curran 2005). Citizen science projects, even with the best of intentions and designs, obviously cannot address these problems.

Another concern are the incentive structures within academia, which are, arguably, quite out of line with the role that academia should play in democratic societies. As many critics (e.g., Sarewitz 2006; Edwards and Siddharta 2017) have noted, temporary jobs and the hunt for grant money often push scientists into a survival mode in which publications in high-ranked journals matter more than *either* fundamental research that the scientists find genuinely interesting and important, *or* research that matters for addressing urgent problems in society. Here, other kinds of reform are needed (and they are indeed on their way in some countries^16^); they would probably also provide scientists with more space to undertake risky, long-term citizen science projects of a kind that might be most fruitful for addressing societal problems.

The second point also follows from the perspective of deliberative systems. This perspective emphasizes the need for a plurality of settings and approaches, instead of relying on a small number of deliberative institutions. It is this division of labor that matters for making sure that all epistemic, ethical, and political goals are fulfilled. Applied to citizen science, this means that not all citizen science projects need to be optimized along all dimensions: some might have stronger epistemic goals, while others give center stage to ethical or democratic goals. The organizers of citizen science projects need to reflect on which aims they can and want to achieve, situating their projects in the broader deliberative system of a society, and decide what to focus on. Instead of starting from their own interests and preferences, it might well make sense to start by reflecting—together with others—about the existing landscape of deliberative and other discourses, and to identify possible gaps, e.g., the lack of knowledge about certain issues that could potentially improve a community's democratic participation.

## Footnotes

1 For various case studies, see e.g., the papers in Hecker et al. (2018a, b) (volume), Part IIA.

2 https://www.citizenscience.org/covid-19/ (last accessed Feb 21st, 2021).

3 https://www.weobserve.eu/weobserve-cop4-sdgs/ (last accessed Feb 21st, 2021).

4 In what follows, we assume that obvious ethical issues in citizen science projects are responsibly administered, e.g., by providing appropriate data protection and avoiding conflicts of interest or exploitative relations. See e.g., Resnik et al. (2015) for an overview of ethical considerations.

5 Democratic theory deals with democratic institutions and practices as objects of study but is also committed to the basic moral foundations of democracy, such as the equal standing of all citizens. This commitment cuts across various methodological and philosophical approaches within democratic theory.

6 This attention to the plurality of deliberative sites, and their various social contexts, is in line with the call by Chilvers and Kearnes to "ecologize participation," which, for them means "recognizing, attending to, and/or mapping the diversities of, and interrelations between, sociomaterial collectives of participation and public involvement that make up wider spaces such as systems, issues, and constitutions" (2019: 359). They criticize early contributions to deliberative theory for having suggested a simplistic ("residual realist") view of the public. But these same points have in fact been recognized by deliberative theorists and they have long moved beyond these earlier conceptions. Chilvers and Kearnes themselves briefly refer to the deliberative systems literature (2019: 359) as part of their broader call for "reconstituting participation" (363–5) by paying attention to broader institutional entanglements and (co-)constitutions of social settings, relations, etc.

7 As Landström (2020, chap. 4) reminds us, however, the notions of “invited” and “uninvited” should not be understood in an essentialist way, and other versions are also possible.

8 This function is particularly interesting when other epistemic communities, e.g., with regard to indigenous or local knowledge, are also involved. See Finn et al. (2018) for reflections.

9 Makuch and Aczel (2018: 403–4) here connect citizen science to the diagnosis of a democratic deficit (drawing on Bultitude 2011) and argue that citizen science can help address this problem.

10 To be sure, this does not hold for projects that do not have immediate political or societal implications, e.g., in astronomy.

11 Of course, this does not mean that the wider—indeed often global—dimension of the problems at hand should be neglected. Often, a purely local perspective is insufficient, because it overlooks the global nature of many challenges (and sidelines the global nature of science and the possibility of collaborating with people across the globe). One possible strategy for connecting “local” and “global” is to frame citizen science projects as part of the effort to reach the UN’s Sustainable Development Goals (SDGs) (see e.g., Fraisl et al. 2020). For discussions of the local/global dimension and the challenges it creates, see also Kimura and Kinchy (2019: 351–2), who emphasize the need for a global perspective, but warn against co-option by certain global players and their notion of “development.” In (2019: 43) they again emphasize the various geographical (and temporal) scales on which citizen science can take place.

12 Science is, of course, a highly exclusive system—but as Dickel and Franzen (2015) discuss, digital technologies can challenge its boundaries and make possible new roles at the boundaries.

13 Interestingly, the concept of “testimonial justice” is used neither by Egert and Allen (2019) nor by Williams and Moore (2019); the latter briefly refer to “hermeneutical injustice” (p. 267), another dimension of epistemic injustice discussed by Fricker, but drawing on “testimonial injustice” seems more straightforward.

14 Cf. Kinchy 2017 for the analysis of a case of anticipatory data collection by citizen science volunteers in order to have data available in case environmental harm materializes.

15 However, social media may offer new possibilities here, especially when it comes to local organizing.

16 E.g., the Dutch “Reward and Recognize” Program that is meant to pluralize the way in which the contributions of researchers are being evaluated. See https://www.nwo.nl/nieuws/kennissector-zet-grote-stap-vooruit-nieuwe-manier-erkennen-waarderen (last accessed Feb 21st, 2021).
